# Antimicrobial Activity of Quercetin: An Approach to Its Mechanistic Principle

**DOI:** 10.3390/molecules27082494

**Published:** 2022-04-12

**Authors:** Thi Lan Anh Nguyen, Debanjana Bhattacharya

**Affiliations:** Biology Department, Franciscan Missionaries of Our Lady University, 5414 Brittany Drive, Baton Rouge, LA 70808, USA; thilananhnguyen@franu.edu

**Keywords:** quercetin, antimicrobial activity, mechanism of action, antibacterial, antifungal, antiviral

## Abstract

Quercetin, an essential plant flavonoid, possesses a variety of pharmacological activities. Extensive literature investigates its antimicrobial activity and possible mechanism of action. Quercetin has been shown to inhibit the growth of different Gram-positive and Gram-negative bacteria as well as fungi and viruses. The mechanism of its antimicrobial action includes cell membrane damage, change of membrane permeability, inhibition of synthesis of nucleic acids and proteins, reduction of expression of virulence factors, mitochondrial dysfunction, and preventing biofilm formation. Quercetin has also been shown to inhibit the growth of various drug-resistant microorganisms, thereby suggesting its use as a potent antimicrobial agent against drug-resistant strains. Furthermore, certain structural modifications of quercetin have sometimes been shown to enhance its antimicrobial activity compared to that of the parent molecule. In this review, we have summarized the antimicrobial activity of quercetin with a special focus on its mechanistic principle. Therefore, this review will provide further insights into the scientific understanding of quercetin’s mechanism of action, and the implications for its use as a clinically relevant antimicrobial agent.

## 1. Introduction

**Figure 1:**
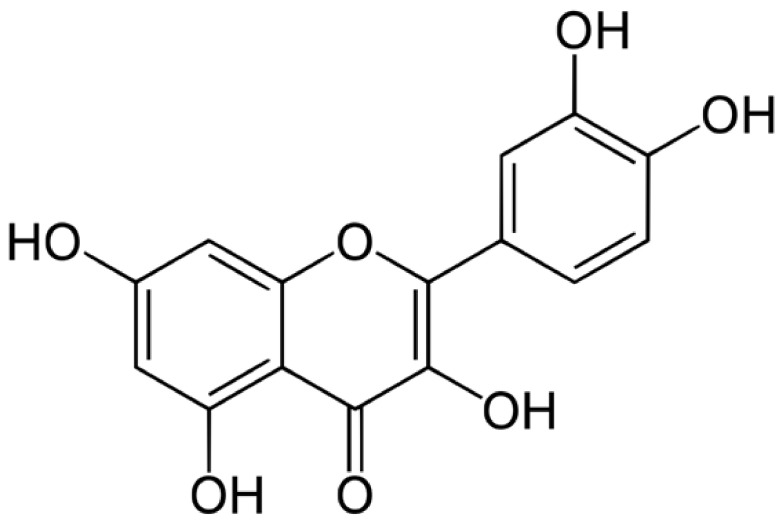
Molecular structure of quercetin.

Quercetin is an important phytochemical, belonging to the flavonoid group of polyphenols. It is widely distributed in various fruits, vegetables, beverages as well as in flowers, leaves, seeds, etc. [1,2,3]. The most abundant source of quercetin is onion (*Allium cepa*), which is one of the most popular vegetables used both as an edible and medicinal plant. Other sources of quercetin include tea, red wine, kales and apples [4]. Many medicinal plants such as *Hypericum perforatum*, *Ginkgo biloba* also contain quercetin [3]. The molecular formula of quercetin is C_15_H_10_O_7_, and its chemical structure is illustrated in Figure 1. Its molecular structure contains a ketocarbonyl group, and the oxygen atom present on the first carbon, being basic, can form salts with acids. Furthermore, a dihydroxy group between the A ring, *o*-dihydroxy group B, C ring C2, C3 double bond, and 4-carbonyl are the active groups present in quercetin. Quercetin’s biological activities have been largely attributed to these active phenolic hydroxyl groups and double bonds [3].

Quercetin possesses many pharmacological activities such as antioxidant [1,5], anticancer, antiviral, antimicrobial, neuroprotection, anti-inflammatory, cardiovascular and anti-obesity [1]. Recently, quercetin has also achieved GRAS (Generally Recognized As Safe) status by the United States Food and Drug Organization [6]. The antimicrobial activity of quercetin has been widely studied by many researchers and has been considered as a potential therapy against different pathogenic microorganisms [1]. In addition, multidrug resistance (MDR) in bacteria has become a major problem worldwide due to the continuous use of antibiotics against bacterial infections. Treatment of these MDR strains has posed a major challenge to the pharmaceutical industry [7]. However, bioactive compounds isolated from different plants, fruits, vegetables, beverages, etc., have gained recent interest in the scientific community due to their beneficial effects in human health [8]. The mechanism of antimicrobial action of these phytochemicals has been extensively investigated currently, in order to utilize them effectively in drug development. Studies on the pharmaceutical properties of quercetin have shown that it can be formulated as a potent natural antimicrobial agent against various pathogenic microorganisms.

In this review, we have summarized the antimicrobial activity of quercetin, with a special focus on its underlying mechanistic principle. Therefore, this review will help future researchers to have a scientific understanding of quercetin’s role in inhibiting microbial diseases as well as its application as a potential antimicrobial agent in the clinical world.

## 2. Antimicrobial Activity

Quercetin is antibacterial against a wide range of bacterial strains, particularly those affecting the gastrointestinal, respiratory, urinary, and integumentary systems [9,10,11]. Quercetin’s antibacterial ability has been linked to its solubility [12] and its interplay with the bacterial cell membrane [13], which is largely determined by the presence of quercetin’s hydroxyl groups [1]. In general, Gram-negative bacteria are more resistant to the bactericidal effects of quercetin than Gram-positive bacteria [14]. The discrepancy in quercetin susceptibility between Gram-positive and Gram-negative bacteria might be partially attributable to the difference in cell membrane composition between the two types of bacteria [1]. However, some quercetin derivatives showed stronger antibacterial ability against Gram-negative than Gram-positive bacteria [1]. Phosphorylation and sulfation of quercetin at different hydroxyl groups were able to enhance or reduce its solubility, and thus altered its antibacterial potential to certain types of bacteria [1,15].

The antibacterial activity of quercetin and quercetin derivatives against different bacterial species occurred at different minimum inhibitory concentrations (MICs). Shu, et al. observed quercetin’s antibacterial effects on the growth of eleven main oral pathogenic microbes: quercetin impeded the growth of six out of the eleven tested microbes including *Streptococcus mutans*, *Streptococcus sobrinus*, *Lactobacillus acidophilu*, *Streptococcus sanguis*, *Actinobacillus actinomycetemocomitans*, *and Prevotella intermedia* with MIC ranging from 1–8 mg/mL [9]. *S. mutans* growth was inhibited on adhesive–dentin interfaces doped with quercetin at MIC of 500 µg/mL [16]. Quercetin also exerted inhibitory effects against *Staphylococcus aureus*, *Pseudomonas aeruginosa* at MIC of 20 mcg/mL [2]. Moreover, quercetin-5,3′-dimethylether exhibited antibacterial effects against *Micrococcus luteus* and *Shigella sonei* at MIC of 25 mcg/mL [17]. Quercetin was also shown to exert antibacterial activities against different Gram-positive bacteria such as Methicillin-resistant *Staphylococcus aureus* (MRSA), Methicillin-sensitive *S. aureus* (MSSA) and Standard *Enterococcus* [18].

In addition, quercetin acted synergistically in combination with other chemotherapeutic compounds and antibiotics to inhibit the growth of bacteria [19,20,21]. When *Pseudomonas fluorescens*, a bacterium implicated in food spoilage, was treated with quercetin along with lactoferrin and hydroxyapatite, the MIC was significantly reduced compared with when the bacteria were treated with quercetin alone [19]. Quercetin further proved its antibacterial ability when working in conjunction with other antibiotics against methicillin-resistant *S. aureus*. [20].

## 3. Antifungal Activity

Although quercetin’s antifungal effects are not as well documented as its antibacterial properties, quercetin has been reported to have antifungal potentials against *Aspergillus fumigatus* (16–64 μM) [21] and *Aspergillus niger* [22].

Quercetin sensitized other drugs against resistant fungal infections [11,23,24]. Oliveira, et al. found quercetin’s antifungal potentials against *Candida albicans* and *Cryptococcus neoformans* to be insignificant. However, when combined with amphotericin B, quercetin significantly enhanced amphotericin B’s antifungal efficacy against *C. neoformans* strains and reduced amphotericin B’s side effects, which Oliveira, et al. explained may be due to quercetin’s antioxidant effects [11]. Similarly, Gao, et al. showed that almost no cell death occurred when fluconazole-resistant *C. albicans* was treated with quercetin alone, while cell death was increased significantly when fluconazole-resistant *C. albicans* was treated with a quercetin–fluconazole combination [23]. Quercetin’s antifungal activity was also observed against *Candida* spp. [25], *C. albicans* and *Saccharomyces cerevisiae* [18].

## 4. Antiviral Activity

Quercetin and its derivatives have long been studied for their antiviral effects. In vitro, quercetin and its derivatives were antiviral against a variety of viruses including human immunodeficiency virus (HIV), poliovirus, Sindbis virus [13], respiratory viruses [13,26,27], and Mayarovirus [28]. In vivo, when administered orally, quercetin offers mice infected with Mengo virus some level of protection against lethal infections [26]. 

In vitro, quercetin enhanced acyclovir’s antiviral ability against herpesviruses [28]. In vivo, when used with vitamin C, quercetin helped prevent and treat patients with early respiratory tract infections, especially including COVID-19 patients [26,29]. A clinical study showed that when treated with Quercetin Phytosome, patients with mild COVID-19 symptoms had shorter time to virus clearance [30]. Quercetin was also found to exert antiviral activity against several serotypes of Rhinovirus, Coxsackievirus (A21 and B1), Poliovirus (type 1 Sabin) and Echovirus (type 7, 11, 12, and 19) [31].

## 5. Mechanism of Antibacterial Activity

### 5.1. Disruption of Bacterial Cell Walls and Cell Membrane

Recent studies have demonstrated that quercetin has the ability to effectively disrupt the integrity of the bacterial cell membrane, thereby inhibiting bacterial growth. Using TEM analysis, Wang, et al. [14] observed that quercetin effectively disrupted the structural integrity of cell wall and cell membrane of *E. coli* and *S. aureus* at concentrations of 50× MIC and 10× MIC, respectively. In treated *E. coli*, the damaged cell wall showed numerous structural abnormalities such as prominent lysis of cell wall, cell distortion, leakage of cytoplasmic materials, cytoplasmic membrane separated from the cell wall, as well as uneven endochylema density. Eventually, cell cavitation and cell death were evident. Similarly, in treated *S. aureus*, significant disruption of the cell wall, thinning of cell membranes, chromatin lysis, leakage of endochylema contents, uneven endochylema density, shedding of extracellular pilli, as well as nuclear cavitation were noted [14]. In this study, the basic mechanism of antibacterial activity was disruption of the permeability of bacterial cell walls and cell membranes, as detected by alkaline phosphatase (ALP) and β-galactosidase activities [14]. Another research group, Zhao et. al. [32], determined potent antibacterial activity of sugarcane bagasse (containing 470 mg quercetin/g polyphenol) extract against *S. aureus*, *E. coli*, *Listeria monocytogenes* and *Salomonella typhimurium*. The primary antibacterial mechanism was found to be disruption of the cell membrane structure and permeability due to an increase in electrical conductivity, thereby leading to leakage of cellular electrolytes. Results showing damage of cellular proteins were also obtained from protein gel electrophoresis. Moreover, abnormalities in cell morphology and damage of internal structures were evident from SEM and TEM images in *E. coli* and *S. aureus* suspensions treated with their respective MICs, as compared with the untreated ones [32].

In another study, quercetin-loaded nanoparticles significantly inhibited the growth of drug-resistant *E. coli* and *Bacillus subtilis*, causing disruption of the cell wall and membrane, as determined by AFM, SEM and TEM analyses [33]. Quercetin also produced growth inhibitory activity against ceftazidime-susceptible *Streptococcus pyogenes*; the main mechanisms being damage to the cytoplasmic membrane, disappearing of peptidoglycan, and distortion of cell shape [34]. Quercetin was also found to disrupt the cell membrane structure and integrity in carbapenem-resistant *Pseudomonas aeruginosa* and *Acinetobacter baumannii* [35] as well as carbapenem-resistant *E. coli* and *Klebsiella pneumoniae* [36].

### 5.2. Disruption of Nucleic Acid Synthesis

Quercetin also plays an important role in inhibiting the synthesis of nucleic acids in bacterial cells. It has been found that quercetin was able to inhibit the function of *E. coli* DNA gyrase, thereby leading to disruption of DNA synthesis [13,37,38]. In a recent study, Plaper, et al. [39] reported that quercetin inhibited the ATPase activity of *E. coli* DNA gyrase enzyme by binding to the submit GyrB. In this study, they determined the enzyme- binding activity of quercetin by measuring the fluorescence of isolated DNA gyrase subunits in both the presence and absence of quercetin. The binding site was found to be overlapped with that of ATP and the antibiotic novobiocin. Quercetin fluorescence was inferred when these compounds were added to the test samples, resulting in a competitive inhibition of quercetin’s activity. Inhibition of ATPase activity of GyrB was also determined by a coupled ATPase assay [39]. Therefore, from these studies, it can be said that quercetin’s ability to inhibit the function of DNA gyrase enzyme is partially responsible for its antibacterial activity.

Furthermore, Liu, et al. [40] predicted that the antibacterial mechanism of quercetin against the growth of *Lactobacillus* spp. and *Bifidobacterium* spp in laying hens was by inhibiting the DNA supercoiling activity of gyrase. This ultimately led to disruption of bacterial DNA replication. These findings were in agreement with the results of Ohemeng, et al. [38] and supported the fact that the inhibition of DNA gyrase activity might be partially responsible for the antibacterial activity of quercetin.

In a recent study [41], it was shown that quercetin in a complex with iron was able to intercalate with DNA and cause significant cleavage of the supercoiled form of DNA into nicked circular form. Here, the antibacterial mechanism of quercetin was attributed to its nuclease activity. Quercetin was also found to bind to bacterial single-stranded DNA (ssDNA)-binding protein (SSB) of *Pseudomonas aeruginosa*, thereby inhibiting bacterial DNA synthesis [42].

### 5.3. Inhibition of Biofilm Formation

Another antibacterial mechanism of quercetin is prevention of biofilm formation by various bacterial species. Quercetin is known to interfere with the pathways involved in bacterial quorum sensing, thereby preventing bacterial adhesion to target organs [3]. In a recent study, quercetin was found to inhibit almost 95% of the biofilm production of *Enterococcus faecalis*, a Gram-positive opportunistic pathogen [43]. In this study, quercetin showed antibacterial activity at sub-MIC values, and its biofilm inhibitory activity was determined by scanning electron microscopy (SEM) and confocal laser scanning microscopy (CLSM). Furthermore, the authors used proteomics analysis and real-time PCR to determine that quercetin interfered with the proper functioning of the proteins involved in glycolytic, protein folding and protein translation–elongation pathways, thereby disrupting cell physiology and preventing biofilm formation [43]. In another study by Dias da Costa Júnior, et al. [44], quercetin displayed anti-biofilm activity and inhibited almost 50% of biofilm production of methicillin- and vancomycin-resistant *S. aureus* and *Staphylococcus saprophyticus* at sub-MIC doses.

Recent studies have demonstrated that quercetin effectively inhibits the quorum sensing mechanism in *P. aeruginosa* primarily by decreasing its biofilm formation, swimming motility and production of various virulence factors such as protease, elastase, pyocyanin [45]. These results also agree with that of Manner, et al. [46]. They have also reported that quercetin was able to significantly inhibit the production of the pigment violacein, an indicator if bacterial quorum sensing in *Chromobacterium violaceum*, thereby affecting the bacterial quorum-sensing mechanism. Quercetin was also found to interfere with the swarming and swimming motility of *P. aeruginosa*, thus inhibiting bacterial biofilm formation and quorum sensing [46]. Recently, it was found that a nanoparticle complex made up of quercetin and chitosan (quercetin-chitosan nanoplex) affected bacterial quorum sensing by significantly inhibiting the biofilm formation and swimming motility of *P. aeruginosa* [47]. The biofilm formation of a common oral pathogen *S. mutans*, which is the main causative agent of caries, was also inhibited by quercetin [48]. Moreover, quercetin inhibited the biofilm formation of drug-resistant *S. aureus* as well as suppressed the expression of bacterial adhesion genes [49]. Quercetin was found to inhibit biofilm production of *L. monocytogenes* by affecting surface colonization and by reducing cell attachment and secretion of extracellular proteins [50,51].

According to Kim, et al. [52], the biofilm production of vancomycin-resistant *Enterococcus faceium* was inhibited by about 70% by a quercetin–pivaloxymethyl conjugate. They also found that this conjugate showed a synergistic effect with several antibiotics such as vancomycin, ampicillin and cefepime against resistant strains of *Staphylococcus* and *Enterococcus* [52]. Quercetin inhibited the biofilm formation of the Gram-positive bacteria *Bacillus subtilis* FB17 [53]. Vanaraj, et al. [54] found that quercetin–silver nanoparticles effectively inhibited quorum sensing by decreasing the production of violacein in the reporter bacterium *C. violaceum* as well as reducing the formation of biofilm and expression of virulence genes in multidrug resistant *Staphylococcus aureus*. In another study by Yang, et al. [16], it was found that quercetin-doped adhesive was able to inhibit the biofilm formation of the *Streptococcus mutans* strain Ingbritt.

Anti-biofilm activity of quercetin was also observed against multidrug resistant *P. aeruginosa* [55], *Streptococcus pneumoniae* D39 by inhibiting the activity of sortase A [56], *P. aeruginosa* by affecting the *lasIR* quorum sensing system via *vfr* [57], *Escherichia coli* O157:H7 and *Vibrio harveyi* [58], *Staphylococcus epidermidis* by downregulating the expression of intercellular adhesion proteins [59] and a variety of Gram-positive and Gram-negative bacteria such as *Bacillus subtilis* NCIB 3610, enteroaggregative *E. coli* (EAEC) strain 55989, *P. aeruginosa* (P. a.) strain PA14, *E. coli* K12 strain AR3110 [60] as well as *Porphyromas gingivalis*, the causative bacterium of periodontal disease [61]. In another study, onion extracts rich in quercetin and its derivatives inhibited the swarming motility and violacein production in *P. aeruginosa* PAO1, and *Serratia marcescens* MG1 [62].

### 5.4. Reduction of Expression of Virulence Factors

Additionally, quercetin possesses the ability to reduce the activities of several essential virulent enzymes, thereby hindering bacterial virulence. From the studies of He, et al. [61], it was observed that quercetin was able to inhibit the virulence factors such as gingipain proteases, haemagglutinin, hemolytic activities of *P. gingivalis* in a dose-dependent manner. According to Wang, et al. [63], quercetin inhibited the activity of coagulase enzyme, an essential virulence factor of *S. aureus*, thereby protecting rats from catheter-related *Stapylococcul* infections. Quercetin in combination with the antibiotic meropenem was found to inhibit the expression of the virulence factors *bla*_NDM_ and *AdeB* in carbapenem-resistant *P. aeruginosa* and *Acinetobacter baumannii* [36]. Furthermore, quercetin–meropenem combination inhibited the expression of the virulence factors bla*_VIM_* and *ompC* in the clinical isolates of *E. coli* and *Klebsiella pneumoniae* belonging to the carbapenem-resistant Enterobacteriaceae family [37].

In a recent study, quercetin was shown to downregulate the expression of virulence genes *sigB*, *prfA*, *inlA*, *inlC*, and *actA* in *L. monocytogenes* [51]. Furthermore, quercetin was found to inhibit the activity of the virulence factor pneumolysin of *Streptococcus pneumoniae* [64].

**Figure 2:**
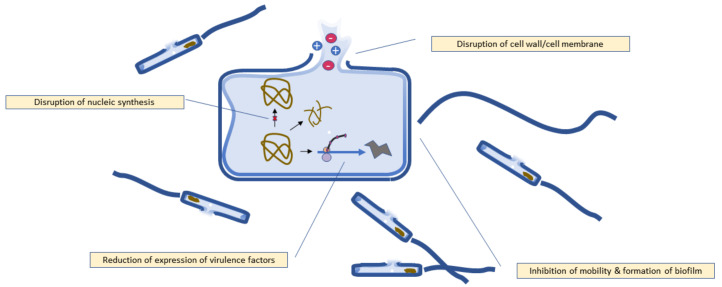
Antibacterial mechanism of quercetin.

The mechanism of antibacterial activity of quercetin is illustrated in Figure 2.

## 6. Mechanism of Antifungal Activity

The antifungal activity of quercetin has also been studied recently. Although little research work has been conducted on its antifungal activity, the mechanisms mainly include disruption of the plasma membrane and inhibition of nucleic acid synthesis, protein synthesis and mitochondrial functions [65]. The recent studies describing quercetin’s antifungal mechanism are summarized in Table 1.

**Figure 3:**
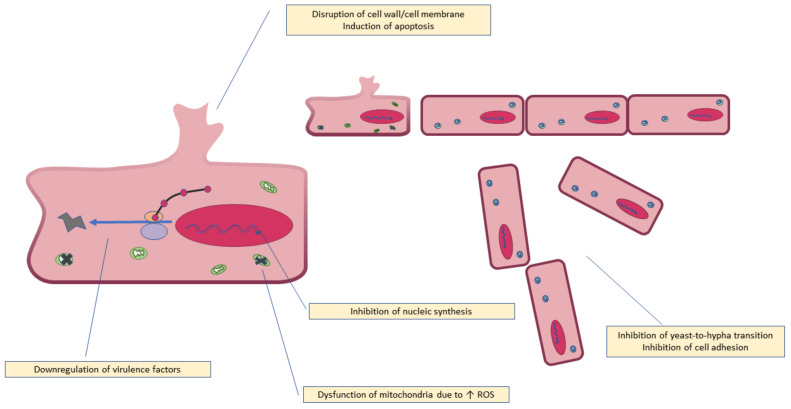
Antifungal mechanism of quercetin.

The mechanism of antifungal activity of quercetin is illustrated in Figure 3.

## 7. Mechanism of Antiviral Activity

Quercetin has been shown to possess significant antiviral properties in recent research works. The primary mechanism of its antiviral activity includes blocking of essential viral enzymes such as polymerases, reverse transcriptase, proteases, integrase, along with suppression of DNA gyrase, and binding viral capsid proteins [13,29,71]. The relevant experiments describing quercetin’s antiviral mechanism are demonstrated in Table 2.

**Figure 4:**
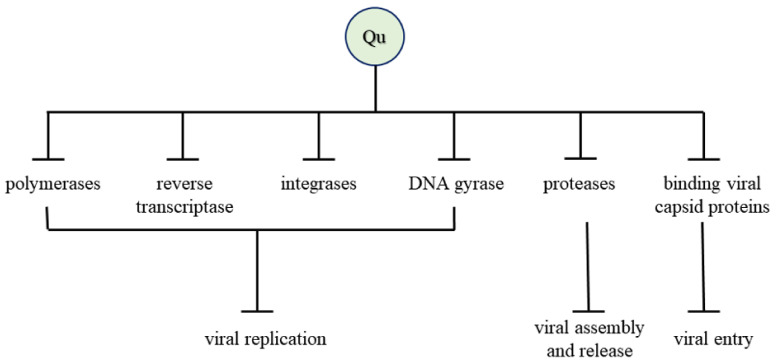
Antiviral mechanism of quercetin.

The antiviral mechanism of quercetin is illustrated in Figure 4.

## 8. Bioavailability of Quercetin

Although quercetin has been reported to have significant antimicrobial activity, it has relatively low bioavailability. This is because it has poor solubility in water, limited permeability, it is unstable in stomach and intestine, and it has relatively short biological half-life [87]. One study showed that free quercetin could not be found in human plasma even after an oral administration of high concentration of quercetin [88]. Phase 2 metabolism significantly affects quercetin’s bioavailability in humans due to the fact that it is extensively metabolized in liver prior to reaching the systemic circulatory system [87,89]. All these factors are responsible for causing low oral bioavailability of quercetin, thereby hindering its applications as a pharmaceutical agent. Therefore, research has been conducted to develop suitable delivery systems that would not only increase the bioavailability of quercetin but also produce significant therapeutic effects, thereby enhancing its biological activities [87]. These delivery systems would protect quercetin molecules in the upper gastrointestinal tract and help in its prolonged release in the colon in order to increase its bioavailability [90].

## 9. Merits and Demerits

Quercetin, an essential plant flavonoid, is well known for its several pharmacological properties. Its broad spectrum antimicrobial activity and mode of action is well studied, which suggests its use as a potent antimicrobial agent in the clinical world. Some of the advantages of using phytochemicals against infectious microorganisms are that these natural compounds cause less side effects to the host compared to antibiotics, and the microorganisms become less drug resistant. However, there are several disadvantages of using these phytochemicals. Sometimes, quercetin was found to be less effective than antibiotics, and its activity increased in combination with antibiotics. In some studies, it was seen that structural modification of quercetin increased its activity significantly versus the parent molecule. Moreover, the oral bioavailability and absorption of quercetin in the human body is poor, which limits its use as a therapeutic agent.

## 10. Conclusions and Future Prospects

From the extensive research works conducted in the past few years, it can be concluded that quercetin exhibits potent antimicrobial activity against a variety of bacteria, fungi and virus. The mechanism of its antimicrobial activity primarily includes disruption of cell membrane integrity, inhibition of nucleic acid synthesis, inhibition of biofilm formation, mitochondrial dysfunction and inhibition of virulence factor expression. Furthermore, the continuous emergence of multidrug resistance in microorganisms has necessitated the search for new antimicrobial agents with a targeted mode of action, causing less side effects. Many phytochemicals isolated from different natural sources have been seen to exhibit therapeutic potentials against a number of different microorganisms; quercetin being one of the most popular and biologically active among them. The existing literature suggests broad-spectrum antimicrobial activities of quercetin, and therefore, it may be considered as a potentially useful antibacterial, antifungal and antiviral nutraceutical. However, its poor oral bioavailability and absorption in the human body hinders its use as an effective antimicrobial agent. Further work is required to enhance its bioavailability in order to exploit its therapeutic potential. Moreover, research in mechanism-based experiments, structural modification of quercetin, and investigation of the interaction between quercetin and its target sites will lead to the design of more potent antimicrobial quercetin derivatives in the future.

## Figures and Tables

**Table 1:** Antifungal mechanism of quercetin.

Fungus Name	Mechanism of Action	References
*Trichophyton rubrum*	Downregulated the enzyme fatty acid synthase and reduced ergosterol levels, thereby causing plasma membrane disruption	[66]
*C. albicans*	Induced apoptosis with increase in intracellular magnesium along with mitochondrial dysfunction. Mitochondrial antioxidant system was disrupted due to increased levels of intracellular ROS and decreased intracellular redox levels. DNA damage was also observed.	[67]
*Cochliobolus lunatus*	Inhibition of nucleic acid synthesis	[68]
*Candida tropicalis*	Induced apoptosis, caused morphological changes, disruption of membrane integrity, increase in intracellular ROS, mitochondrial depolarization and DNA damage in combination with the antibiotic fluconazole.	[69]
*C. albicans*	When combined with fluconazole, quercetin inhibited biofilm formation by downregulating the expression of biofilm-forming genes. The combination also inhibited cell adhesion, cell surface hydrophobicity (CSH), flocculation, fungal metabolism, yeast-to-hypha transition.	[23]
*C. albicans*	Downregulated virulence factors such as biofilm formation, hemolytic activity, activities of the enzymes, proteinase, phospholipase, and esterase, as well as hyphal development. Quercetin in combination with fluconazole induced fungal cell death by apoptosis.	[24]
*Candida parapsilosis* complex	Inhibited biofilm formation	[70]

**Table 2:** Antiviral mechanism of quercetin.

Virus Name	Mechanism of Action	References
Human Immunodeficiency Virus (HIV)-1 strain	Inhibited the enzyme integrase	[72]
Herpes Simplex Virus (HSV), Poliovirus, Respiratory Syncytial Virus (RSV), Sindbis virus	Inhibited viral polymerase and binding of viral capsid proteins or viral nucleic acid	[13]
HSV-1	Reduced infectivity, intracellular replication	[73]
Polio-virus type 1	Reduced infectivity, intracellular replication	[73]
Parainfluenza virus type 3 (Pf-3)	Reduced infectivity, intracellular replication	[73]
RSV	Reduced infectivity, intracellular replication	[73]
Influenza A H1N1	Inhibited neuraminidase	[74]
Influenza H7N9	Inhibited neuraminidase	[75]
Hepatitis C virus (HCV)	Inhibited nonstructural protein 3 (NS3) of HCV helicase	[76]
HCV genotypes 3 and 4	Inhibited the function of p7 proteins	[77]
HCV	Inhibited NS3 protease	[78]
HCV	Downregulated diacylglycerol acyltransferase (DGAT)	[79]
HSV-1	Blocked viral binding and viral penetration to the host cell as well as inhibited the activation of NF-κB at the beginning of infection.	[80]
HSV-2	Blocked viral binding and viral penetration to the host cell as well as inhibited the activation of NF-κB at the beginning of infection.	[80]
Acyclovir-resistant HSV-1	Blocked viral binding and viral penetration to the host cell as well as inhibited the activation of NF-κB at the beginning of infection.	[80]
Influenza A Virus (PR8)	Reduced replication, induced the secretion of type I interferon (IFN) and other pro-inflammatory cytokines in vitro	[81]
Vesicular Stomatitis Virus (VSV)	Reduced replication, induced the secretion of type I interferon (IFN) and other pro-inflammatory cytokines in vitro	[81]
HSV	Reduced replication, induced the secretion of type I interferon (IFN) and other pro-inflammatory cytokines in vitro	[81]
Newcastle Disease Virus (NDV)	Reduced replication, induced the secretion of type I interferon (IFN) and other pro-inflammatory cytokines in vitro	[81]
Influenza A subtypes (H1N1, H5N2, H7N3, and H9N2)	Reduced replication, induced the secretion of type I interferon (IFN) and other pro-inflammatory cytokines in vivo	[81]
Dengue virus type-2 (DENV-2)	Inhibited replication, reduced the levels of ribonucleic acid (RNA)	[82]
Influenza virus	Inferred with viral replication by blocking endocytosis, inhibiting the activity of phosphatidylinositol 3-kinase, inhibiting RNA polymerase and other proteins, increasing antiviral response of mitochondria.	[83]
Influenza A viruses (IAVs)	Inhibited the activity of hemagglutinin	[84]
Dengue virus	Phosphorylation of NS3	[85]
Singapore grouper iridovirus (SGIV)	Interfered with viral binding to target host cells	[86]

## Data Availability

Not applicable.
